# Synergistic antitumor and immunomodulatory effects of *Bifidobacterium animalis* subsp. *lactis* V9 combined with anti–PD-1 therapy

**DOI:** 10.3389/fimmu.2026.1791276

**Published:** 2026-05-26

**Authors:** Liuqing Yang, Zelong Li, Yalin Li, Qi Zhang, Heping Zhang

**Affiliations:** 1 Key Laboratory of Dairy Biotechnology and Engineering, Ministry of Education, Inner Mongolia Agricultural University, Hohhot, Inner Mongolia, China; 2 Key Laboratory of Dairy Products Processing, Ministry of Agriculture and Rural Affairs, Inner Mongolia Agricultural University, Hohhot, Inner Mongolia, China; 3 Inner Mongolia Key Laboratory of Dairy Biotechnology and Engineering, Inner Mongolia Agricultural University, Hohhot, Inner Mongolia, China; 4 Department of Colorectal Surgery, Zhejiang Cancer Hospital, Hangzhou, Zhejiang, China

**Keywords:** antineoplastic, anti–PD-1 therapy, *Bifidobacterium animalis* subsp. *lactis* V9, gut microbiota, immune modulation

## Abstract

**Background:**

Immune checkpoint inhibitors (ICIs) targeting PD-1/PD-L1 have revolutionized cancer immunotherapy but remain limited by low response rates, immune-related adverse events (irAEs), and reduced efficacy following antibiotic exposure. The gut microbiota critically influences ICI responsiveness, and *Bifidobacterium* species have emerged as potent immunomodulatory commensals. However, the mechanistic contribution of specific live biotherapeutic strains remains unclear.

**Methods:**

We systematically characterized *Bifidobacterium animalis* subsp. *lactis* V9 (*B. lactis* V9), through in-vitro cytokine assays and multiple syngeneic tumor models (CT26, MC38, 4T1). Immunophenotyping, microbiota colonization, and toxicological studies were conducted to evaluate efficacy, immune modulation, colonization stability, and safety.

**Results:**

*B. lactis* V9 dose-dependently induced TNF-α, IL-6, and IL-10 secretion in THP-1 macrophages, exhibiting a balanced cytokine profile distinct from LPS stimulation. *In vivo, B. lactis* V9 alone moderately inhibited tumor growth but synergized with αPD-1 to achieve a 50% tumor growth inhibition and extend survival in CT26 models, accompanied by increased IFN-γ^+^CD8^+^ T cells and activated CD86^+^CD11c^+^ dendritic cells. The synergy persisted despite antibiotic pretreatment, indicating colonization stability (8×10^8^–10^9^ copies/g) and a metabolite-driven mechanism. In 4T1 models, B. lactis V9 co-therapy mitigated αPD-1–induced uterine inflammation and pulmonary hemorrhage by downregulating IL-1α, IL-1β, and IL-17A while maintaining effector cytokines. Toxicology assessments revealed no adverse findings up to 3.52×10^12^ CFU/kg (acute) or 5.00×10^11^ CFU/kg (90-day repeated dose), with all genotoxicity tests negative.

**Conclusions:**

*B. lactis* V9 harmonizes pro- and anti-inflammatory responses through TLR2/TLR4–NF-κB/MAPK signaling, remodels the tumor microenvironment, and enhances αPD-1 efficacy without increasing toxicity. Its colonization resilience and favorable safety profile support clinical translation as a microbiome-based adjunct to immunotherapy. These results provide a mechanistic foundation for combining live biotherapeutic products with ICIs to optimize antitumor immunity.

## 1 Introduction

The global burden of malignant tumors continues to rise. In 2018, there were 1.93 million new cases and 940,000 deaths from colorectal cancer, and 2.26 million new cases of breast cancer, both representing major solid tumor types (1–3). In China, colorectal and breast cancers rank second and fourth in incidence, respectively, and the 5-year survival rate of advanced-stage patients remains below 30%, underscoring the urgent need for improved therapeutic strategies (3–5).

Immune checkpoint inhibitors (ICIs), represented by anti–PD-1 antibodies (αPD-1), have achieved a major breakthrough in solid tumor therapy by blocking the PD-1/PD-L1 pathway and relieving T-cell immunosuppression (4, 5). However, their clinical application remains limited due to several challenges. The objective response rate (ORR) is low, reaching only 10–25% in colorectal and breast cancers, and less than 5% in microsatellite-stable (MSS) subtypes (6, 7). In addition, the incidence of immune-related adverse events (irAEs) is high, with grade 3–4 toxicities occurring in 15–20% of patients, often leading to treatment discontinuation or even fatal outcomes (8–10). Furthermore, antibiotic exposure markedly reduces the efficacy of αPD-1 therapy; approximately 20–30% of patients receiving broad-spectrum antibiotics experience decreased response rates and shortened survival (11, 12).

The gut microbiota is increasingly recognized as a “hidden immune organ,” playing a pivotal role in regulating tumor immune responses and modulating the efficacy of ICIs (13, 14). Mechanistically, beneficial bacteria such as Bifidobacterium and Akkermansia promote dendritic cell (DC) maturation, upregulate CD86 and MHC-II expression, and enhance CD8^+^ T-cell infiltration and IFN-γ production (15–17). In addition, short-chain fatty acids (SCFAs) and inosine regulate immune activity via histone deacetylase (HDAC) inhibition and GPR109a activation, thereby enhancing CD8^+^ T-cell function and suppressing Treg differentiation (18–21). Moreover, the microbiota strengthens the intestinal barrier by upregulating tight junction proteins such as occludin and ZO-1, reducing lipopolysaccharide (LPS) translocation, and mitigating inflammation-mediated suppression within the tumor microenvironment (TME) (22, 23). Collectively, microbial composition and metabolic activity critically influence host immune balance and represent key modulators of ICI responsiveness.

Live biotherapeutic products (LBPs), as a novel form of microbiota-based intervention, offer advantages over conventional probiotics, including defined indications, regulatory oversight, and mechanistically interpretable effects (21, 24). Studies have shown that Bifidobacterium-based LBPs can markedly enhance ICI efficacy; in melanoma models, they potentiate αPD-L1 antitumor effects through DC-dependent activation of CD8^+^ T cells (15, 25, 26), thereby providing a scientific rationale for LBP–ICI combination therapy. *Bifidobacterium animalis* subsp. *Lactis* V9 (*B. lactis* V9), the bacterial strain investigated in this study, isolated from the feces of healthy children. It possesses multiple advantageous features. It exhibits immunostimulatory properties by inducing DC maturation through TLR2/4 activation, promoting IL-12 secretion, and suppressing IL-17A, which collectively enhance CD8^+^ T-cell cytotoxicity (27–29). It also demonstrates strong colonization capacity, tolerating gastric acid and bile salts, and adheres to intestinal epithelial cells via extracellular polysaccharides (EPS) to sustain its immunological effects (30). Furthermore, it shows excellent safety; the strain has been applied in food production, and toxicological studies confirm its safety at high oral doses and its ability to alleviate antibiotic-associated dysbiosis (24).

Although Bifidobacterium species have been demonstrated to augment ICI efficacy, comprehensive studies on *B. lactis* V9 remain lacking (17, 31). Its synergistic mechanism with αPD-1, colonization stability and therapeutic persistence following antibiotic pretreatment, and its effects on irAEs, long-term safety, and dosage windows have not yet been elucidated (25, 32, 33). Therefore, this study systematically characterizes the immunological and pharmacological properties of *B. lactis* V9, focusing on its dose-dependent activation of THP-1 macrophages, its synergistic effects with αPD-1 and remodeling of the TME in CT26, MC38, and 4T1 models, its colonization and mitigation of irAEs following antibiotic pretreatment, and its acute, repeated-dose, and genotoxicity profiles to define a safe therapeutic window for clinical translation.

## 2 Materials and methods

### 2.1 Animal experiments

#### 2.1.1 Study design and oversight

All animal procedures were performed in accordance with the Guidelines for Care and Use of Laboratory Animals of Inner Mongolia Agricultural University (Permit No: SYXK-2020-0002) and were approved by the Animal Ethics Committee of Inner Mongolia Agricultural University (No: NND2023105). The studies were designed to evaluate, both *in vitro* and *in vivo*, the efficacy, immunological effects, colonization, and safety of *B. lactis* V9, a live biotherapeutic product (LBP). Animals were randomized into treatment groups when tumor volumes were comparable, and investigators were blinded to treatment assignments during tumor measurement, histological assessment, and flow cytometric analysis.

#### 2.1.2 Bacterial strain and preparation

**Figure 1:**
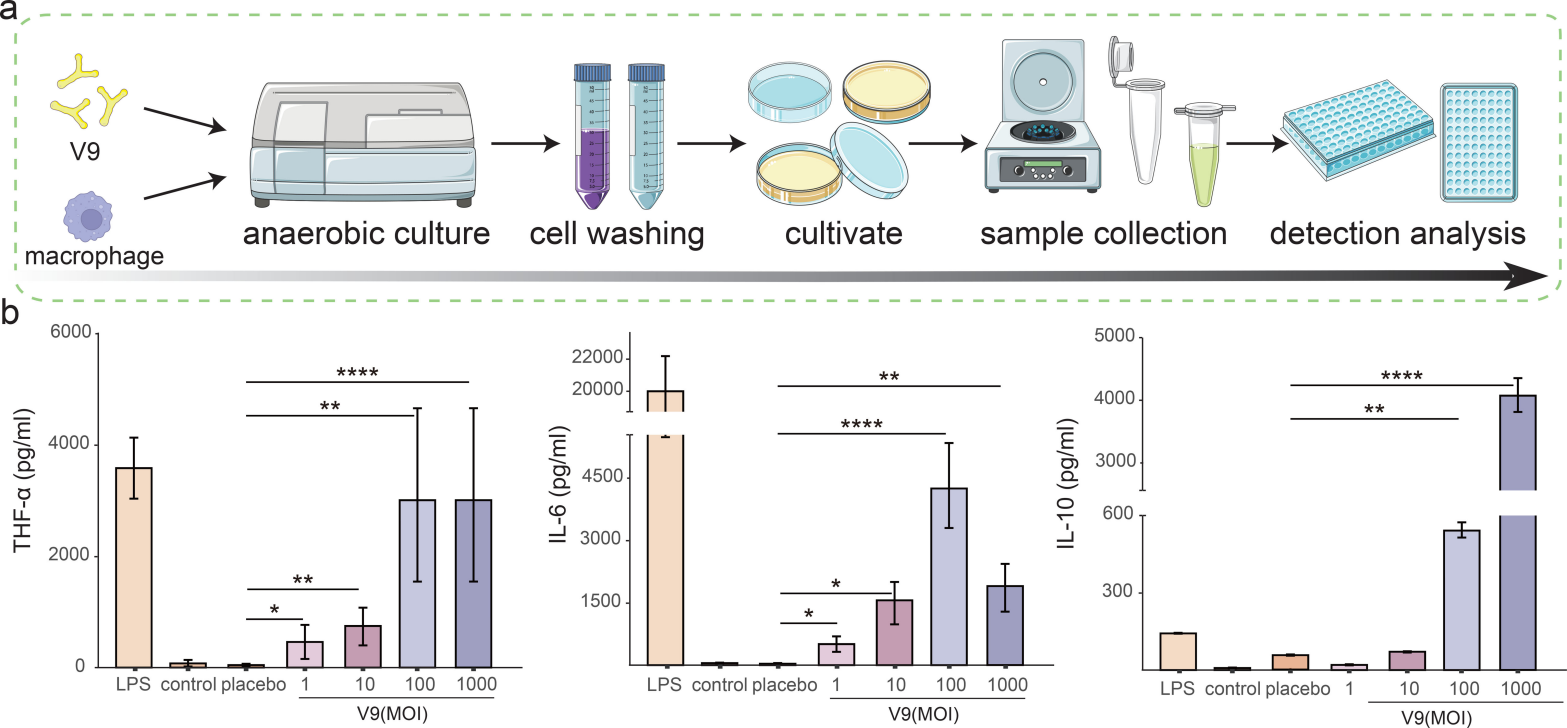
*B. lactis* V9 Induces Immune Activation *In Vitro*. **(a)** Experimental workflow for evaluating cytokine responses in THP-1–derived macrophages. **(b)** Levels of cytokines TNF-α, IL-6, and IL-10 at different dose gradients. LPS; positive control; Control: blank control. *P < 0.05; **P < 0.01, ***P < 0.0001; Kruskal–Wallis test.

*B. lactis* V9 originally isolated from the feces of a healthy child and preserved as a master cell bank (MCB). The working strain was produced by Jinhua Galaxy Biotechnology Co., Ltd. Cultures were grown anaerobically (85% N_2_;/10% CO_2_;/5% H_2_;) in a custom-modified MRS-based medium at 37 °C in 5000 L fermenters until the late-log phase (OD_600_ > 18, variation < 1 OD/h). Cells were harvested by centrifugation (4000 rpm, 15 min, 4 °C), washed twice, and resuspended in sterile saline containing 10% skim milk and 10% sodium glutamate as cryoprotectants. The resulting lyophilized powder contained 8.0×10¹^0^–8.0×10¹¹ CFU/g. Viability (≥8.0×10¹^0^ CFU/g), identity (16S sequencing and strain-specific qPCR), purity (aerobic plating, mycoplasma PCR), and endotoxin levels (<5 EU/mL, LAL assay) were confirmed before use (**Figure 1a**).

#### 2.1.3 Cell lines and culture

CT26 (murine colon carcinoma, BALB/c origin), MC38 (murine colon adenocarcinoma, C57BL/6 origin), 4T1 (murine mammary carcinoma, BALB/c origin), and THP-1 (human monocytic leukemia) cell lines were obtained from the American Type Culture Collection (ATCC, Manassas, VA, USA). All lines were authenticated by short tandem repeat (STR) profiling and confirmed to be mycoplasma-negative before use. CT26 and 4T1 were maintained in RPMI-1640, MC38 in DMEM, and THP-1 in RPMI-1640, each supplemented with 10% heat-inactivated fetal bovine serum (FBS; Gibco, USA) and 1% penicillin–streptomycin. For bacterial co-incubation assays, antibiotics were omitted from the culture medium.

#### 2.1.4 Animals and tumor models

Female BALB/c and C57BL/6 mice (6–8 weeks, 18–22 g) were obtained from Shanghai Lingchang Biotechnology Co., Ltd. (Shanghai, China). Animals were maintained under specific pathogen-free (SPF) conditions with a 12 h light/dark cycle at 20–24 °C and 40–60% humidity, receiving autoclaved chow and water ad libitum. CT26 model: BALB/c mice were subcutaneously inoculated with 5×10^5^ CT26 cells in 100 µL sterile PBS into the right flank. MC38 model: C57BL/6 mice received 1×10^6^ MC38 cells in 100 µL PBS. 4T1 model: BALB/c mice were injected with 1×10^5^ 4T1 cells in 50 µL PBS into the axilla. Tumor growth was measured every 2–3 days using calipers, and tumor volume was calculated as (length × width²)/2.

#### 2.1.5 Constructing different tumor models

CT26 model: SPF BALB/c mice were divided into three groups for the experiment. Mice were subcutaneously inoculated with CT26 cells (5 × 105 cells) on day 0 (D0). In Group 1 (concurrent administration group), mice received daily oral gavage of *B. lactis* V9 (1×1010 CFU/mouse/day) from D7 to D20. Anti–PD-1 antibody (clone RMP1-14, Bio X Cell, NH, USA; 200 µg/mouse, intraperitoneally) was administered on D8, D11, D14, and D17 (1-day interval between *B. lactis* V9 and anti–PD-1 treatment). In Group 2 (short-interval group), mice were gavaged with *B. lactis* V9 at the same dose from D7 to D20; anti–PD-1 was administered on D14, D17, D20, and D23 (7-day interval between the initiation of *B. lactis* V9 and anti–PD-1 treatment). In Group 3 (pre-treatment group), mice received daily *B. lactis* V9 gavage from D0 to D23. CT26 cells were inoculated on D7, and anti–PD-1 antibody was administered on D14, D17, D20, and D23 (14-day interval between the onset of *B. lactis* V9 administration and the first anti–PD-1). Each experimental arm included vehicle controls and single-agent controls (*B. lactis* V9 alone or anti–PD-1 alone), with n = 8 per group. Tumor volume was measured every for 2–3 days, and body weight and general health status were monitored throughout the study.

MC38 model: SPF C57BL/6 mice were acclimatized for one week. On day 1, MC38 cells (containing 1×10^6^ cells) were subcutaneously inoculated in the right axilla of the mice. On day 11 (mean tumor volume 95 mm3), the mice were randomly divided into four groups: control group, *B. lactis* V9 group, αPD-1 group, and *B. lactis* V9 +αPD-1 group (n = 8 per group). From day 11 to day 22, mice were continuously administered *B. lactis* V9 (5 x 10^7^ CFU/d) by gavage, and αPD-1 (200 μg/d) was injected intraperitoneally on days 12, 15, 18, and 20.

4T1 model: SPF C57BL/6 mice were acclimatized for one week. On day 1, 4T1 cells (1×10^5^ cells) were subcutaneously injected into the right axilla of each mouse. On day 11 (mean tumor volume 95 mm3), mice were randomly divided into four groups: control group, *B. lactis* V9 group, αPD-1 group, and *B. lactis* V9 +αPD-1 group (n = 8 per group). From day 11 to day 22, mice were continuously administered *B. lactis* V9 (2 x 10^9^ CFU/d) by gavage, and αPD-1 (100 μg/d) was injected intraperitoneally on days 12, 15, 18 and 20.

### 2.2 *B. lactis* V9 colonization experiment

Mice were pretreated for two weeks with an antibiotic cocktail administered in drinking water, consisting of ampicillin (1 g/L), neomycin (1 g/L), metronidazole (1 g/L), vancomycin (0.5 g/L), and amphotericin B (0.1 g/L). On day 1, mice were subcutaneously inoculated with MC38 cells. On day 2, animals were orally gavaged with *B. lactis* V9 at doses of 1×10^8^, 1×10^9^, or 1×10¹^0^ CFU/day for 20 consecutive days. Anti–PD-1 treatment was initiated on day 11. Fecal samples were collected 24 hours after the final gavage, and *B. lactis* V9 abundance was quantified by Quantitative Real-time PCR (qPCR). In the non-antibiotic control experiment, the 2-week antibiotic pretreatment was omitted; mice were gavaged with *B. lactis* V9 at 2×109 CFU/day starting on day 2, and all subsequent procedures were identical to those described above.

### 2.3 *In vitro* cytokine assays

THP-1 cells were differentiated into macrophages using phorbol 12-myristate 13-acetate (PMA; 100 ng/mL) for 48 h, followed by a 24 h rest in PMA-free medium. Cells (2×10^5^ per well, 24-well plates) were exposed to *B. lactis* V9 at multiplicities of infection (MOI) of 1, 10, 100, or 1000. After 2 h of co-incubation, cells were washed and incubated for an additional 22 h in fresh medium. Supernatants were collected, clarified, and analyzed using the LEGENDplex™ Human Th Cytokine Panel (BioLegend, San Diego, CA). Lipopolysaccharide (LPS; 1 µg/mL) served as a positive control. Each assay included n = 3 biological replicates, analyzed in duplicate.

### 2.4 Flow cytometry

Tumors were excised and digested at 37 °C for 60 min in RPMI containing collagenase I (1 mg/mL; Sigma-Aldrich), DNase I (50 µg/mL; Roche), and gentamicin (50 ng/mL), then filtered through a 70 µm strainer to obtain single-cell suspensions. After Fc blocking, cells were stained with Zombie Aqua viability dye, anti-CD8 (clone 53-6.7, FITC), anti-CD11c (clone N418, APC), and anti-CD86 (clone GL-1, PE; all from BioLegend). For intracellular cytokine staining, cells were restimulated with PMA (50 ng/mL), ionomycin (1 µg/mL), and brefeldin A (5 µg/mL) for 4 h, then fixed, permeabilized, and stained with anti–IFN-γ (clone XMG1.2, PerCP-Cy5.5). Data were acquired using a BD FACSCanto II cytometer and analyzed with FlowJo v10.7 (BD Biosciences).

### 2.5 Plasma cytokine profiling

At the experimental endpoint, blood was collected by cardiac puncture into EDTA tubes, centrifuged at 2000×g for 10 min at 4°C, and plasma stored at −80 °C until analysis. Cytokine concentrations (IL-1α, IL-1β, IL-6, IL-12p70, IL-17A, IL-23, IL-27, MCP-1, TNF-α, IFN-β, IFN-γ, GM-CSF) were quantified using the LEGENDplex™ Mouse Inflammation Panel (BioLegend) according to the manufacturer’s instructions.

### 2.6 Histopathology

After the experiment, the heart, liver, spleen, lungs, kidneys and uterus of 4T1 model mice were collected and fixed in 10% neutral-buffered formalin, embedded in paraffin, sectioned at 4 µm, and stained with hematoxylin and eosin (H&E). Slides were evaluated under a Nikon Eclipse Ci microscope by a board-certified pathologist blinded to treatment, and tissue injury was scored on a semiquantitative 0–4 scale.

### 2.7 Colonization assay

Fecal samples were collected 24–72 h after the final *B. lactis* V9 administration and stored at −80 °C. DNA was extracted using a QIAamp Fast DNA Stool Mini Kit (Qiagen, Germany). *B. lactis* V9 abundance was quantified by strain-specific qPCR using primers *qB. lactis* V9-F11 (CATCTGGTTTCTCCTTTCAGGTTTG) and *qB. lactis* V9-R11 (GATGCCATATCTCAATCTGCTCATT). Standard curves were generated from serial dilutions of *B. lactis* V9 genomic DNA (10¹–10^7^ copies). Results were expressed as gene copies per gram of feces and validated by comparison with plate counts to confirm the copies-to-CFU conversion.

### 2.8 Statistical analysis

Tumor growth curves were analyzed by two-way repeated-measures ANOVA with Tukey’s *post hoc* test. End-point tumor weights and immune-cell frequencies were compared using one-way ANOVA or the Kruskal–Wallis test with Dunn’s correction for nonparametric data. Cytokine levels were corrected for multiple comparisons using the Benjamini–Hochberg false discovery rate (FDR). Statistical analyzes were performed using GraphPad Prism v9.0 (GraphPad Software, San Diego, CA) and R v4.2. A P value < 0.05 was considered statistically significant.

## 3 Results

### 3.1 *B. lactis* V9 induces immune activation *in vitro*

We evaluated the immunostimulatory effects of *B. lactis* V9 on macrophages *in vitro*. Macrophages were co-cultured with gradient concentrations of *B. lactis* V9 at different multiplicities of infection (MOIs), with lipopolysaccharide (LPS) serving as a positive control. Cytokine levels of TNF-α, IL-6, and IL-10 in the culture supernatants were quantified. Compared with the control group, *B. lactis* V9 induced a dose-dependent increase in IL-10 and TNF-α secretion. As the MOI increased from 1 to 1000, the levels of TNF-α and IL-10 increased significantly (P < 0.01; **Figure 1b**). Notably, IL-6 production peaked at an MOI of 100, followed by a decline at MOI 1000, although its concentration remained significantly higher than that of the control group (P < 0.01; **Figure 1b**). Collectively, these findings demonstrate that *B. lactis* V9 effectively activates macrophages and triggers robust cytokine responses, upregulating both pro-inflammatory (TNF-α, IL-6) and anti-inflammatory (IL-10) mediators.

### 3.2 *B. lactis* V9 enhances antitumor efficacy of αPD-1 in colorectal tumor models

**Figure 2:**
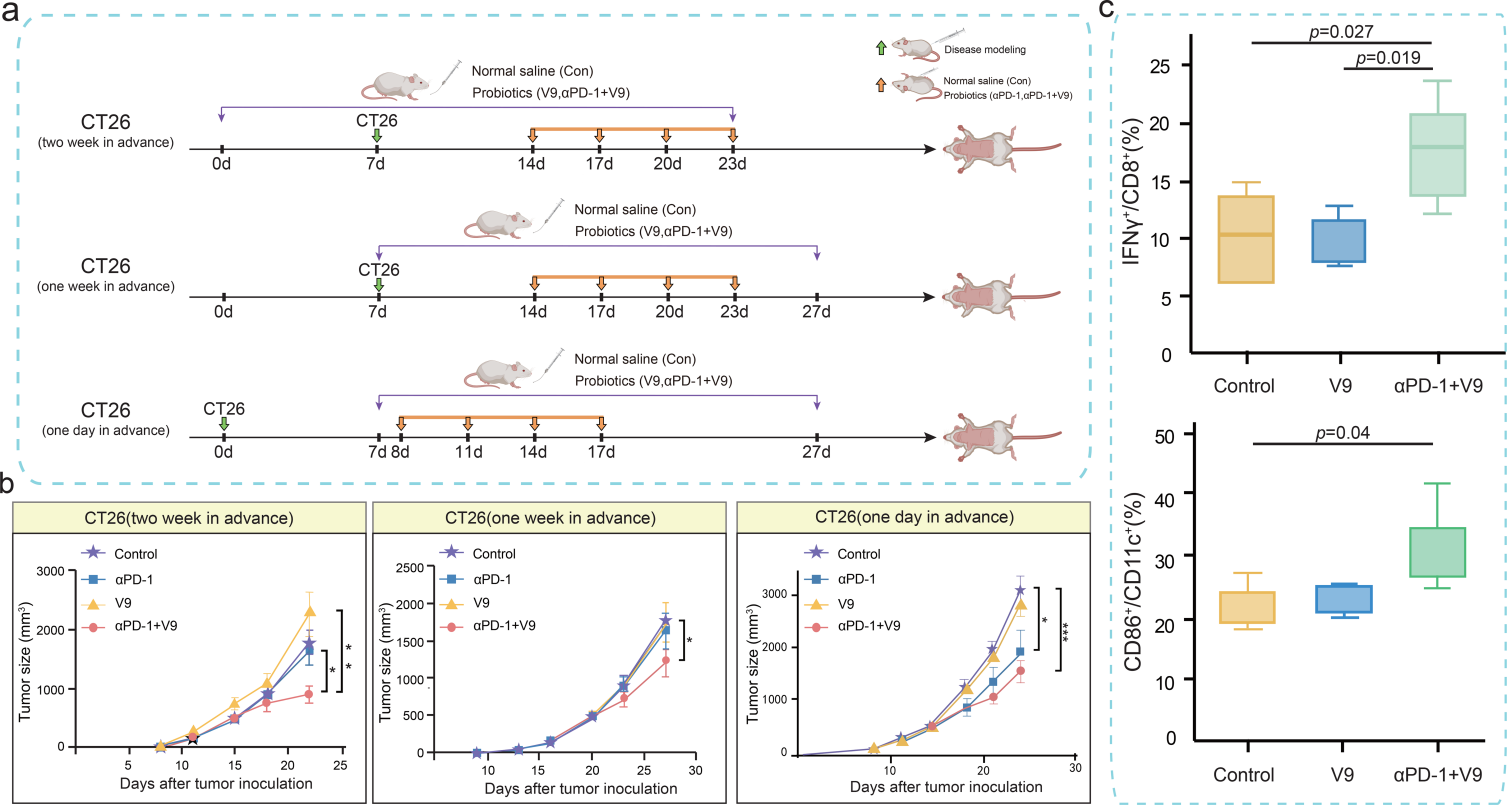
Antitumor effects of different *B. lactis* V9 pretreatment times in a mouse colon tumor model. **(a)** Animal experiment schedule and dosing regimen. **(b)** Trends in tumor volume changes in mice under different antibiotic administration times. **(c)** CD8+ and CD86+/CD11c+ in tumors of mice in different groups n = 8 per group (*P* < 0.05; Kruskal–Wallis test).

A mouse colon cancer model was established using CT26 cells, and tumor volume was measured periodically. The experiment was divided into three groups, receiving *B. lactis* V9 intervention 2 weeks (Group 1), 1 week (Group 2), and 1 day (Group 3) before αPD-1 administration, respectively, followed by αPD-1 treatment during the intervention period. We observed that tumor volume increased over time in all groups. Regardless of treatment duration, the *B. lactis* V9 group exhibited limited antitumor activity, but significantly enhanced the immunotherapy effect when combined with αPD-1 therapy. The synergistic effect with αPD-1 increased to some extent with prolonged pretreatment time. At the experimental endpoint, the tumor volume of mice in the combined treatment group was significantly lower than that in the control group (Group1: 895.32 ± 98.65 mm3 vs 1875.56 ± 152.14 mm3; Group2: 1252.45 ± 118.32 mm3 vs 1885.67 ± 178.45 mm3; Group3: 1493.21 ± 148.56 mm3 vs 3086.78 ± 245.32 mm3; mean ± SE; P < 0.05; **Figure 2b**). Notably, mice that started the intervention two weeks earlier had significantly lower tumor volumes than the other single-treatment groups (895.32 ± 98.65 mm3 vs 1786.41 ± 145.78 mm3; mean ± SE; P < 0.05; **Figure 2b**). Further analysis of the flow cytometry results revealed that the proportion of IFNγ^+^CD8^+^ T cells in the combined intervention group was significantly higher than that in the control group and the *B. lactis* V9-only group (P = 0.027, P = 0.019; **Figure 2c**). Simultaneously, the proportion of CD86^+^CD11c^+^ dendritic cells was significantly higher in the combined intervention group than in the control group and the *B. lactis* V9-only group (P = 0.04), indicating that the combined treatment may enhance the function of cytotoxic T cells, promote the maturation of dendritic cells, and enhance antigen presentation capacity.

### 3.3 *B. lactis* V9 enhances the anti-tumor effect of αPD-1

**Figure 3:**
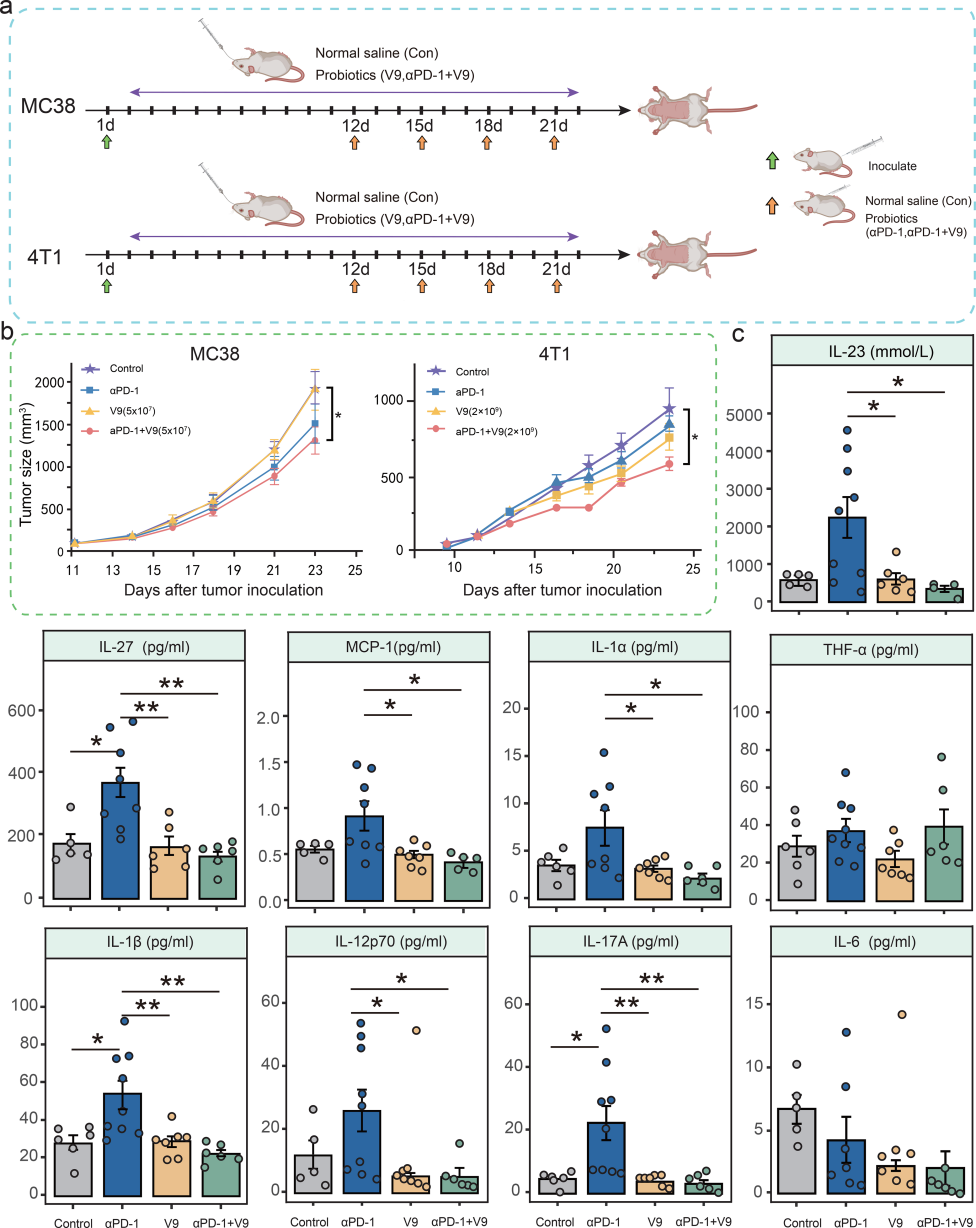
Antitumor efficacy of *B. lactis* V9 in mouse models of breast and colon cancer. **(a)** Animal experiment schedule and dosing regimen. **(b)** Trends in tumor volume changes in different groups of mice. **(c)** Serum cytokine levels in different groups of mice n = 8 per group (*P* < 0.05; Kruskal–Wallis test). *P<0.05; **P<0.01.

To evaluate the antitumor effects of *B. lactis* V9, mouse models of colorectal cancer (MC38) and breast cancer (4T1) were established. Tumor growth was monitored every other day from day 12 to day 22. As expected, tumors in all groups exhibited progressive enlargement over time, and *B. lactis* V9 alone did not suppress tumor growth. However, mice receiving the combination of *B. lactis* V9 and αPD-1 showed consistently smaller tumor volumes compared with the control group. At the experimental endpoint, tumor sizes were significantly reduced in the combination group in both models (MC38: 1341.62 ± 166.05 mm³ vs. 1870.37 ± 237.10 mm³; 4T1: 587.77 ± 49.21 mm³ vs. 912.34 ± 26.99 mm³; mean tumor volume ± SEM; P < 0.05; **Figure 3b**). Notably, although the combination therapy also resulted in smaller tumors compared with αPD-1 monotherapy (1263.73 ± 238.27 mm³ vs. 1512.44 ± 312.53 mm³),but the difference was not statistically significant (P > 0.05). These results indicate that *B. lactis* V9 combined with αPD-1 therapy showed a trend of slowing tumor growth in both tumor models, suggesting that it may enhance the anti-tumor effects of immune checkpoint inhibitors.

Serum cytokine profiles were assessed across treatment groups using a multiplex immunoassay. No significant differences were observed in IL-6 or TNF-α levels among the groups (P > 0.05; **Figure 3c**). Compared with the control group, αPD-1 significantly increased the levels of several pro-inflammatory cytokines, including IL-1β, IL-17A, and IL-27, while both *B. lactis* V9 alone and the combination of *B. lactis* V9 with αPD-1 attenuated these αPD-1-induced elevations, restoring cytokine levels toward those of the control group. Interestingly, *B. lactis* V9 alone or in combination with αPD-1 also significantly reduced the levels of multiple pro-inflammatory mediators such as IL-1α, IL-23, IL-1β, and IL-27 (P < 0.05; **Figure 3c**). These findings indicate that *B. lactis* V9 administration does not induce systemic immune activation; instead, it mitigates excessive inflammatory responses induced by αPD-1 treatment, thereby potentially enhancing the safety profile of immune checkpoint blockade therapy.

**Figure 4:**
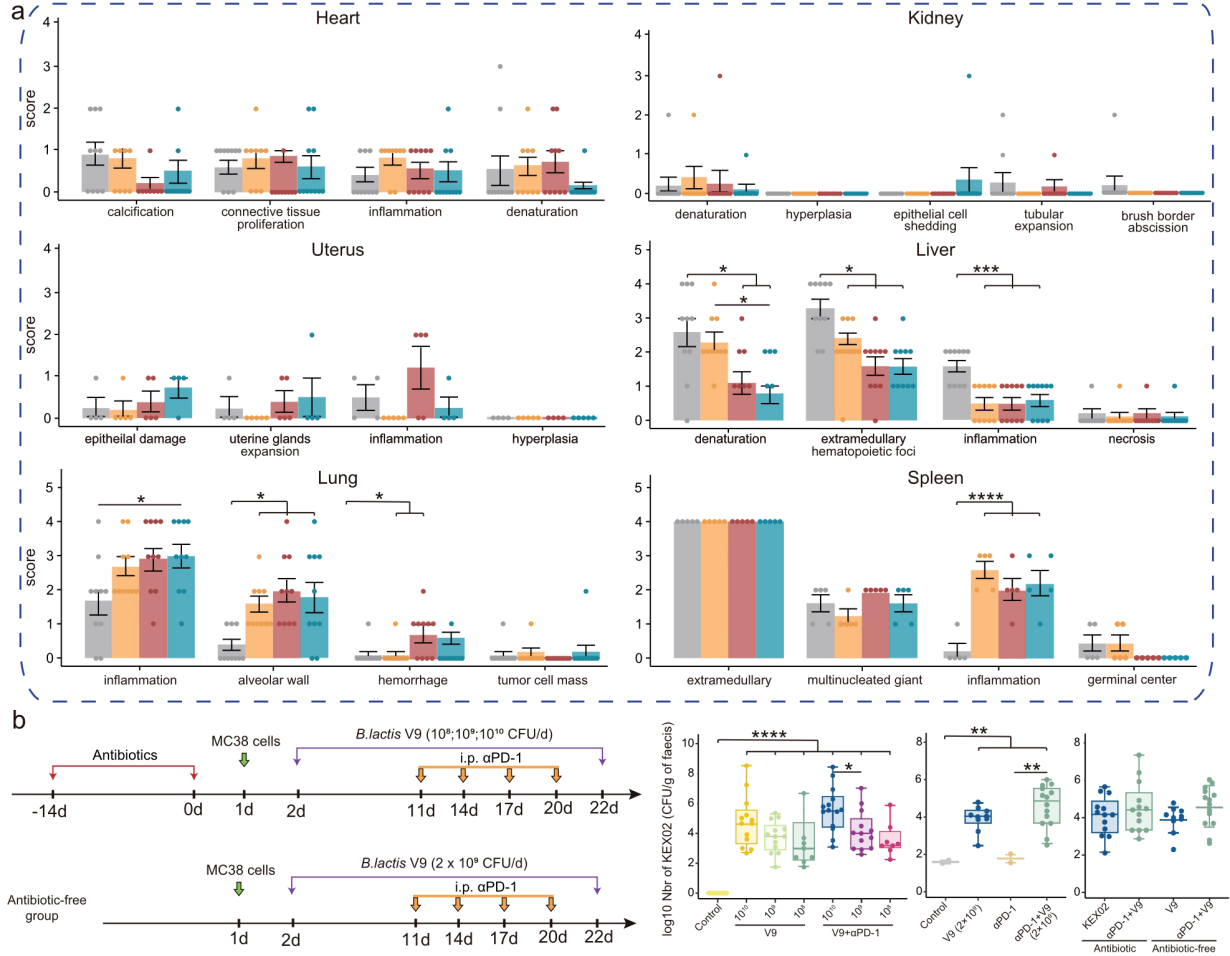
The effects of antibiotic treatment on the colonization stability and cytotoxicity of KEX02. **(a)** Injury scores of different organs in mice after intervention. **(b)** Analysis of bacterial abundance in mouse feces by qPCR; data are presented as log_10_-transformed colony-forming units (CFU) per gram of feces. **(c)** Efficacy of *B. lactis* V9 in combination with αPD-1 across various tumor models. n=10 per group; *P < 0.05; **P < 0.01, ***P < 0.0001, ****P<0.0001.

Histopathological evaluation showed that *B. lactis* V9, either alone or in combination with anti-PD-1 therapy, did not induce detectable pathological alterations in the heart, kidneys, or uterus of 4T1 tumor-bearing mice. In contrast, *B. lactis* V9 treatment ameliorated liver degeneration, inflammatory infiltration, and extramedullary hematopoiesis, indicating a protective effect against liver injury. A similar pattern was observed in the spleen, where mild inflammatory changes were noted, which may reflect immune activation. Importantly, the combination of *B. lactis* V9 and anti-PD-1 did not exacerbate pulmonary lesions; instead, *B. lactis* V9 mitigated αPD-1–induced pulmonary hemorrhage (**Figure 4a**). These findings suggest that *B. lactis* V9 exerts a favorable safety profile and may alleviate specific immune-related tissue damage associated with checkpoint blockade therapy.

### 3.4 Antibiotic pretreatment does not alter intestinal colonization of *B. lactis* V9

To evaluate whether antibiotic pretreatment affects the intestinal colonization of *B. lactis* V9 and its potential synergistic antitumor activity, mice were administered antibiotics for two weeks and then gavaged with *B. lactis* V9 at 1×10^8^, 1×10^9^, or 1×10¹^0^ CFU/day for 20 days. qPCR analysis showed that antibiotic-treated mice exhibited *B. lactis* V9 levels of 10³–10^6^ CFU/g, with fecal abundance positively correlated with the gavage dose (P < 0.05; **Figure 4b**). In tumor-bearing mice without antibiotic treatment, daily gavage of 1×10^9^ CFU for 20 days resulted in fecal *B. lactis* V9 levels of 10^4^–10^5^ CFU/g. Importantly, the fecal *B. lactis* V9 abundance did not differ significantly between antibiotic-treated and untreated mice (P > 0.05; **Figure 4b**), indicating that antibiotic pretreatment does not have a decisive impact on *B. lactis* V9 colonization in the gut.

## 4 Discussion

*B. lactis* V9 upregulates TNF-α, IL-6, and IL-10 in THP-1 macrophages in a dose-dependent manner and, compared with LPS, yields a more balanced cytokine profile. This phenotype is consistent with reports describing TLR2- and EPS–TLR2-mediated regulation of the NF-κB/MAPK pathways, enabling a dynamic equilibrium between pro- and anti-inflammatory responses (18, 23, 34). Unlike LPS, which primarily activates TLR4, *B. lactis* V9 may induce a “balanced M1/M2” polarization via TLR2/TLR4 crosstalk, thereby constraining excessive inflammation (17, 35–37). Such bidirectional regulation provides a safety foundation for *in vivo* application and aligns with reports that Bifidobacterium enhances ICI efficacy through the TLR2–IFN-γ axis without inducing excessive toxicity (38, 39).

*In vivo*, the antitumor efficacy of *B. lactis* V9 appears to rely on modulation of the immune microenvironment (14, 40). Specifically, we observed that pre-treatment with *B. lactis* V9 for different durations (2 weeks, 1 week, or 1 day) prior to αPD-1 therapy consistently suppressed CT26 tumor growth. This effect was accompanied by a marked increase in intratumoral IFN-γ^+^ CD8^+^ T cells with potent effector functions, as well as enhanced activation of antigen-presenting cells, such as CD86^+^ CD11c^+^ dendritic cells. These findings suggest that *B. lactis* V9 may facilitate the transition of tumors from an immunologically “cold” to a “hot” phenotype, potentially through early remodeling of the gut microecosystem and EPS-mediated dendritic cell activation that strengthens antigen presentation (15, 41, 42). Consistent with our observations, Gao et al. reported that although the probiotic strain Probio-M9 alone did not significantly alter tumor burden, its combination with anti-PD-1 therapy markedly suppressed tumor progression. This combined effect was associated with improved gut microbial composition, increased infiltration and activation of cytotoxic T lymphocytes, and reduced regulatory T-cell activity within the tumor microenvironment, thereby enhancing PD-1 blockade–mediated tumor killing (42). Similar immunomodulatory mechanisms have been described for several probiotic strains, including Bifidobacterium spp., Lactobacillus johnsonii, and Lacticaseibacillus rhamnosus GG. These strains promote dendritic cell engagement at the intestinal mucosa, facilitate antigen presentation and immune activation, and subsequently traffic through mesenteric lymph nodes to tumor-draining lymph nodes, ultimately augmenting immune checkpoint blockade–induced T-cell infiltration (15, 25, 43, 44).

Although microbe-based interventions have shown considerable promise in enhancing the efficacy of immune checkpoint blockade (ICB), their antitumor activity as monotherapies is generally limited (40). In both the MC38 and 4T1 mouse tumor models, *B. lactis* V9 alone failed to markedly suppress tumor progression, suggesting that *B. lactis* V9 by itself may be insufficient to overcome the profoundly immunosuppressive tumor microenvironment. This observation is consistent with the nature of many probiotic-based therapies, whose primary roles involve immune modulation and systemic priming rather than direct cytotoxicity (45–47). Notably, the combination of *B. lactis* V9 with anti–PD-1 therapy resulted in a pronounced synergistic antitumor effect, implying that *B. lactis* V9 may function as an “immune sensitizer.” By reshaping host immune status or modulating the local metabolic landscape, *B. lactis* V9 may create a microenvironment that is more permissive to PD-1 blockade. Previous studies have shown that Clostridium butyricum surface proteins can bind receptor proteins on cancer cells, inhibit the GRP78 and PI3K–AKT–NF-κB pathways, modulate multiple cytokines, suppress cytotoxic T lymphocytes, and promote tumor-associated macrophage formation—highlighting the diverse mechanisms through which microbial components influence tumor immunity (48, 49). Histopathological analyzes further strengthen the therapeutic relevance of *B. lactis* V9. Whether administered alone or together with anti–PD-1, *B. lactis* V9 did not induce detectable pathological abnormalities in the heart, kidneys, or uterus. Instead, *B. lactis* V9 alleviated hepatic degeneration, inflammatory infiltration, and extramedullary hematopoiesis, while inducing only mild, immunologically interpretable inflammatory changes in the spleen. Moreover, *B. lactis* V9 mitigated anti–PD-1–associated pulmonary hemorrhage. Collectively, these findings not only support the preliminary safety profile of *B. lactis* V9 but also suggest a potential protective role against ICB-induced tissue injury.

Antibiotics are indispensable to modern medicine, yet their broad activity inevitably perturbs the resident gut microbiota, reducing both diversity and richness and potentially compromising therapies that rely on host–microbiome interactions, such as immune checkpoint blockade (50, 51). Therefore, assessing whether a probiotic adjuvant can successfully colonize the gut following antibiotic exposure is critical for evaluating its translational potential. In this study, fecal qPCR analysis revealed that despite two weeks of broad-spectrum antibiotic pretreatment, *B. lactis* V9 exhibited a clear dose-dependent increase in intestinal abundance (10³–10^6^ CFU/g). Notably, its overall colonization level remained comparable to that of mice without antibiotic exposure, indicating that *B. lactis* V9 is capable of establishing itself within a heavily perturbed gut ecosystem. This resilience may stem from intrinsic biological attributes—such as partial antibiotic tolerance, niche-occupying capacity, or rapid growth kinetics—that enable *B. lactis* V9 to persist or rapidly reestablish following ecological disruption. Interestingly, antibiotic pretreatment did not diminish the colonization or synergistic antitumor activity of *B. lactis* V9 This finding contrasts with clinical observations in which broad-spectrum antibiotics markedly impair ICI efficacy, suggesting that the immunomodulatory benefits of *B. lactis* V9 may rely more on strain-specific metabolic outputs rather than on extensive interactions with the surrounding microbial community. Moreover, higher gavage doses translated into proportionally greater intestinal exposure, which in principle could amplify its ability to potentiate immunotherapy (11, 52, 53). These interpretations, however, should be viewed with caution. Antibiotic class, dosage, spectrum of activity, and treatment timing all shape the trajectory of microbiota recovery and may differentially influence the engraftment of exogenous strains (54). The precise molecular determinants underlying the colonization robustness of KEX02—whether linked to resistance genes, biofilm-forming potential, or specialized metabolic adaptations—remain unknown.

Despite providing initial evidence supporting both the safety and potential synergistic effects of *B. lactis* V9 in combination with immune checkpoint blockade, several limitations of this study should be acknowledged. First, although multiple murine tumor models were employed, inherent differences in tumor evolution, immune system composition, and gut microbiota structure between mice and humans may constrain the direct translation of these findings to clinical applications. Second, as a gut-acting microbial‐derived agent, the causal pathways linking *B. lactis* V9 to enhanced immunotherapy responses remain insufficiently defined. While this study suggests that *B. lactis* V9 may mitigate immune-related tissue injury and modulate host immunity, mechanistic insights into how it influences gut microbial metabolism, host metabolic reprogramming, and tumor immune microenvironment remodeling are still lacking. Comprehensive multi-omics approaches will be necessary to delineate the specific molecular and metabolic axes through which *B. lactis* V9 exerts its immunomodulatory effects. Third, at the clinical level, larger and well-designed prospective trials are required to determine whether *B. lactis* V9 combined with anti–PD-1 therapy can improve objective response rates, progression-free survival, and other clinically meaningful outcomes. Parallel monitoring of dynamic changes in patients’ gut microbiota will also be essential to validate its utility as a microbiome-based immunotherapy adjuvant.

In conclusion, *B. lactis* V9 augments anti–PD-1 efficacy in colorectal and breast cancer models and mitigates immune-related toxicity by harmonizing innate immune activation and remodeling the tumor microenvironment. Its stable colonization after antibiotic exposure and favorable high-dose safety further support its translational promise as a microbiome-based adjunct to immunotherapy. These findings provide both strain-level and mechanistic underpinnings for LBP–ICI combination strategies and lay a foundation for the clinical translation of “gut microbiota modulation of tumor immunity.

## Data Availability

The original contributions presented in the study are included in the article/supplementary material. Further inquiries can be directed to the corresponding author.
